# A novel frameshift mutation in *TRPV6* is associated with hereditary pancreatitis

**DOI:** 10.3389/fgene.2022.1058057

**Published:** 2023-01-09

**Authors:** Idrees A. Shah, Hari Prasad, Sanghita Banerjee, Reuben Thomas Kurien, Sudipta Dhar Chowdhury, Sandhya S. Visweswariah

**Affiliations:** ^1^ Department of Molecular Reproduction, Development and Genetics, Indian Institute of Science, Bangalore, India; ^2^ Department of Gastroenterology, Christian Medical College Vellore, Vellore, Tamil Nadu, India

**Keywords:** pancreatitis, *CaSR*, *FGF23*, WES - whole-exome sequencing, *TRPV6* (transient receptor potential cation channel subfamily V member 6)

## Abstract

**Introduction:** Hereditary pancreatitis (HP) is a rare debilitating disease with incompletely understood etio-pathophysiology. The reduced penetrance of genes such as PRSS1 associated with hereditary pancreatitis indicates a role for novel inherited factors.

**Methods:** We performed whole-exome sequencing of three affected members of an Indian family (Father, Son, and Daughter) with chronic pancreatitis and compared variants with those seen in the unaffected mother.

**Results:** We identified a novel frameshift mutation in exon 11 of *TRPV6* (c.1474_1475delGT; p.V492Tfs*136), a calcium channel, in the patients. Functional characterization of this mutant *TRPV6* following heterologous expression revealed that it was defective in calcium uptake. Induction of pancreatitis in mice induced *Trpv6* expression, indicating that higher expression levels of the mutant protein and consequent dysregulation of calcium levels in patients with chronic pancreatitis could aggravate the disease.

**Discussion:** We report a novel frameshift mutation in TRPV6 in an Indian family with HP that renders the mutant protein inactive. Our results emphasize the need to expand the list of genes used currently for evaluating patients with hereditary pancreatitis.

## Introduction

Hereditary pancreatitis (HP) is a rare familial genetic disorder that is characterized by recurrent episodes of pancreatitis commonly presented in childhood or early adulthood (Shelton and Whitcomb, 2018). These repeated episodes lead to irreversible morpho-physiological changes, including fibrosis, pancreatic exocrine insufficiency, and diabetes mellitus (Ramsey et al., 2017). Moreover, patients with HP have a ∼50 fold risk of developing pancreatic ductal adenocarcinoma (PDAC) (Rebours et al., 2008). This disease is more common in Caucasians and rare among Asians (Shelton and Whitcomb, 2016).

The involvement of a fine protease–antiprotease balance in the pathogenesis of pancreatitis has been a predominant school of thought so far (Hegyi and Sahin-Tóth, 2017). Inactive precursor forms of digestive proteases secreted by pancreatic acinar cells are flushed from the ductal system in a sodium bicarbonate–rich fluid into the duodenum. However, gain-of-function or mutations that cause misfolding of cationic trypsinogen (PRSS1) lead to its premature intrapancreatic activation, causing autodigestion of the pancreas and pancreatitis (Mayerle et al., 2019). Moreover, an increase in calcium concentrations and compartmental distribution in the acinar cells is a known risk factor for acute pancreatitis (Lee and Papachristou, 2019). Dysregulated Ca^2+^ levels can cause premature activation of PRSS1 (Krüger et al., 2000), endoplasmic reticulum stress (Sah et al., 2014), or hampered zymogen granule trafficking in the acinar cells (Messenger et al., 2014) resulting in pancreatitis.

Since the pioneering discovery in 1996 of *PRSS1* mutation in patients with HP (Whitcomb et al., 1996), other mutations in the Cystic Fibrosis Transmembrane Conductance Regulator (*CFTR*), Serine Protease Inhibitor Kazal type 1 (*SPINK1*), Chymotrypsin C (*CTRC*), and Carboxypeptidase A1 (*CPA1*) have been associated with the development of HP (Raphael and Willingham, 2016; Mayerle et al., 2019). Copy number variations (CNV) in *PRSS1* have also been reported in hereditary (LaRusch et al., 2012) and chronic pancreatitis (Chen et al., 2008). However, the reduced penetrance of the mutations in *PRSS1* and the absence of other known pathogenic mutations in pancreatitis susceptibility genes in some affected individuals (Masamune, 2014) suggest the role of additional yet-undiscovered inherited factors. Moreover, the marked variation in the age of disease onset, associated complications, and known genetic predisposition, suggests the complex nature of the disease as an orchestration of gene-gene and gene-environment interactions (Hasan et al., 2018).

**FIGURE 1:**
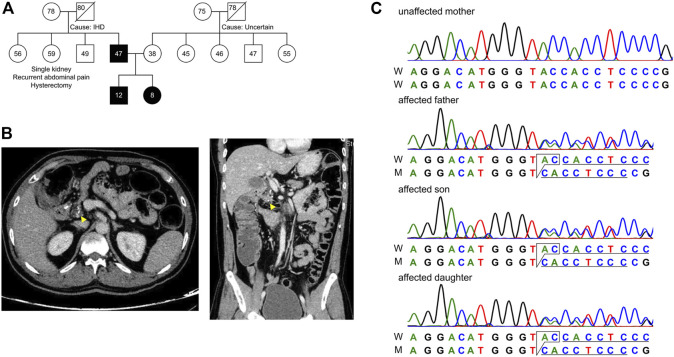
Pedigree of the subjects in this study. **(A)** Extended family pedigree of the subjects. Circle represents females, square represents males and numbers represent age of the individual. Affected trio Father (F), Son (S) and Daughter (D) are marked black, and Mother (M) is unaffected. Except for the Father, none of the first-degree relatives had developed pancreatitis from both paternal and maternal sides of patient S and D. **(B)** CT scan of the abdomen showing transverse (left) and longitudinal (right) sections, with focal calcium deposition (yellow arrow) in the tail of the pancreas of patient F. **(C)** Chromatogram representing c.1474_1475delGT in the affected trio as a heterozygous state.

In the current study, we studied an Indian family with autosomal dominant, hereditary pancreatitis in two generations, the Father (F) and his two children Son (S) and Daughter (D) (Figure 1A). Whole-exome sequencing and CNV analysis in the affected trio revealed a novel, functionally relevant mutation in *TRPV6* (Transient Receptor Potential Vanilloid subfamily member 6).

## Results

### Clinical characterization of the patients

A family with a history of chronic calcific pancreatitis and recurrent pancreatitis in two generations was investigated for an underlying genetic cause. The family pedigree and clinical features of the affected trio are presented in Figure 1A and Table 1. Patient F (Father) had a history of recurrent episodes of abdominal pain since the age of 12. During the episodes the pain was located in the upper abdomen with radiation to the back, lasting for 2–3 days, and occurred at 6–12 months intervals. At the age of 27 he was diagnosed with chronic calcific pancreatitis and underwent a lateral pancreaticojejunostomy. The recent computed tomography (CT) scan of Patient F, now a diabetic, shows evidence of chronic calcific pancreatitis in the head of the pancreas (Figure 1B).

**TABLE 1:** Clinical history of the affected patients (Father (F), Son (S), and Daughter (D).

Medical history	Father	Son	Daughter
Age (years)	47	14	10
First pain episode (at the age of)	12	5	3
Diabetes Mellitus	Yes	No	No
Calcium mg/dL (Normal: 8.3–10.4 mg/dL)	9.18	9.76	9.24
Phosphorous mg/dL (Normal range-2.5–4.6 mg/dL)	3.2	5.6	5.6
Triglyceride (m/dL)- (Normal range -<150 mg/dL)	73	89	55
Vitamin D (ng/mL) (Normal: >30 ng/mL)	12.9	ND	13.6
HbA1C (%)	9.1	5.3	5.2
Fecal Elastase 1 (μg)	<5.5	235	500
CT abdomen/USG	CT: Thin pancreas with calcifications in the head of the gland	USG: Mild thinning of pancreatic body	USG: GB: No sludge
		MOD: not dilated	MPD: 2.2 mm diameter
			Atrophic pancreatic tail
ARFI (m/s) (Normal range: 1.00 ± .17 m/s)	ND	1.32	ND
MRCP	ND	Normal MPD anatomy	Prominent and normal MPD anatomy
Surgery	Lateral Pancreatico-jejunostomy	Nil	Nil

ND, not done; MPD, main pancreatic duct; USG, ultrasonography; ARFI, acoustic radiation force impulse; MRCP, magnetic resonance cholangiopancreatography.

The other two affected members of the family, Son (S) and Daughter (D) reported the first episode of pancreatitis at the age of 3 and 5, respectively. Patient S has suffered eight episodes of acute pancreatitis each more than 6 months apart while patient D suffered multiple episodes of acute pancreatitis. CT of abdomen and endoscopic ultrasonography (EUS) of the patient S did not reveal any features of chronic pancreatitis, however, Acoustic Radiation Force Impulse Imaging (ARFI) of the pancreas revealed an increased shear wave velocity in the pancreas, suggesting pancreatic fibrosis (Sanjeevi et al., 2020). An ultrasound abdomen of patient D showed an atrophic tail of the pancreas and a prominent main pancreatic duct (2.2 mm), suggestive of chronic pancreatitis. While patients F had a history of occasional alcohol use from the age of 21, an evaluation for an etiology for recurrent acute pancreatitis in the affected trio did not reveal any specific cause.

The Mother (M) of the patient S and D does not have any history of pancreatic disorder or diabetes mellitus. No other first degree relative of the primary patient F had developed pancreatitis (Figure 1A). Patients had low serum Vitamin D levels and children presented moderately elevated serum phosphorous levels (Table 1).

### Whole exome sequencing identifies novel mutation in *TRPV6*

To identify genes predisposing the subjects to hereditary pancreatitis, we performed WES on the affected trio–father, son, and daughter, and the unaffected mother. Out of the 44,322 variants called by WES, 710 novel and/or rare missense variants with gAD MAF score of ≤.01 (Karczewski et al., 2020), present in the affected trio and not in the mother were processed further for bioinformatic analysis. Interestingly, we found no mutations that passed quality control (see Materials and Methods) or the expected segregation pattern, in genes known to be associated with pancreatitis such as *PRSS1*, *CFTR*, *SPINK*, *CPA1*, *CTRC*, *CLDN2*, and *PLINP*.

Copy number variant analysis identified a copy gain of the initial segment of the *CBS* gene (58kb, POS: 44475162–44533170, GRCh37). No CNVs in the trio were found in genes hitherto known to be associated with pancreatitis.

**FIGURE 2:**
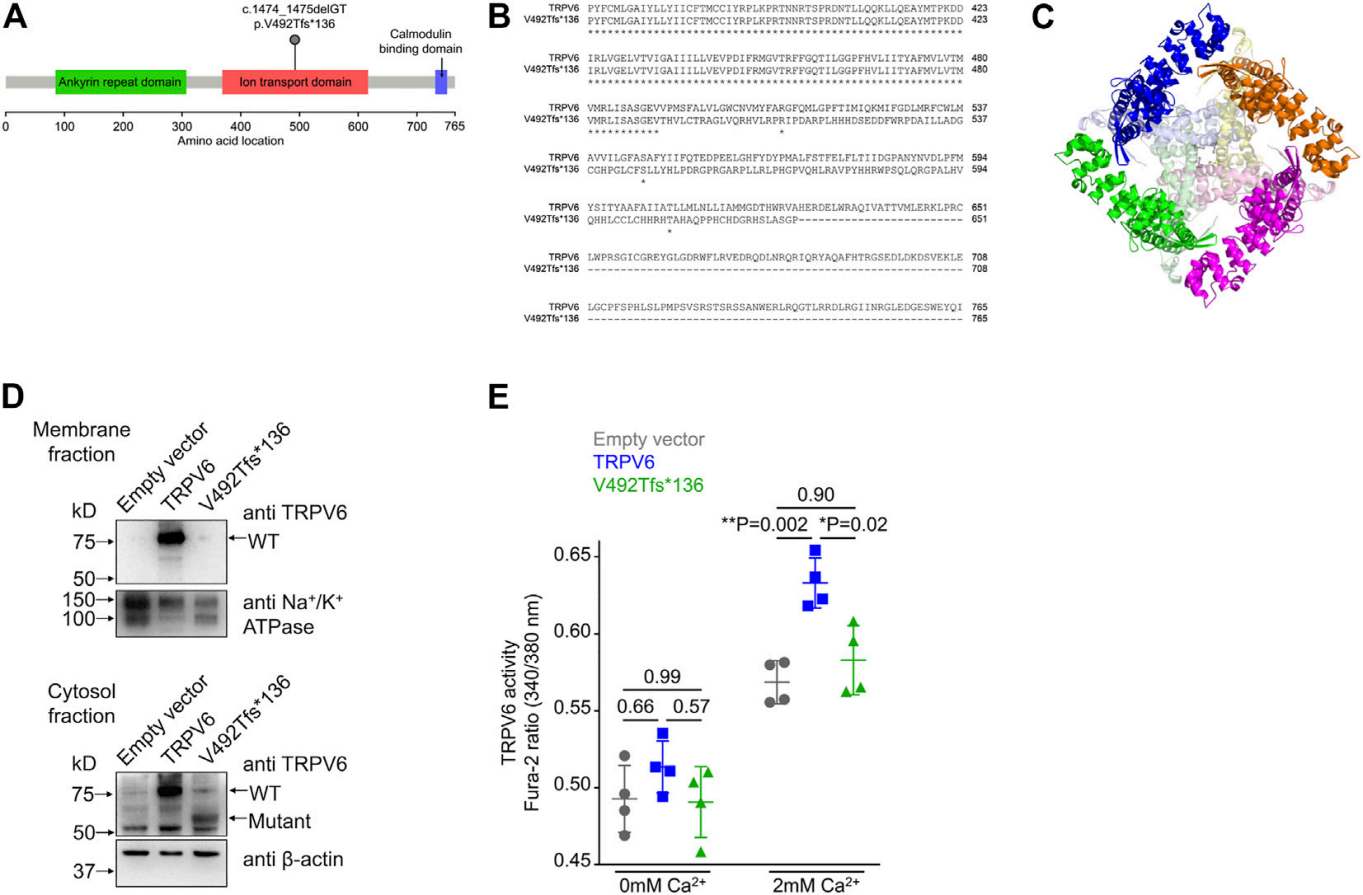
Frameshift mutation in *TRPV6*. **(A)** Diagrammatic representation of the location of the p.V492Tfs*136 mutation in the ion transport domain of *TRPV6*. **(B)** Sequence alignment of wild type and mutant *TRPV6* from amino acid 367. Protein sequences of *TRPV6* were obtained by translating *in silico* the mRNA sequences using *expasy.org* and alignment performed at *embnet.vital-it.ch*. The alignment shows that the mutant protein is predicted to be 626 amino acids long and there is no sequence similarity between the wild type and the mutant protein beyond amino acid 492. **(C)** The structure of *TRPV6* (PDB ID: 6BO8). Individual chains comprising the native tetramer are shown in magenta, green, orange and blue. The regions beyond amino acid 492 in each chain are shown in lighter shades of pink, light green, pale yellow and light blue, respectively. The mutant protein loses the central part of the oligomer which constructs the pore. **(D)** Western blot of membrane (top) and cytosolic (bottom) fractions prepared from HEK 293T cells transfected with either the empty vector, or plasmid harboring either the wild type *TRPV6* or mutant *TRPV6* cDNA. An antibody recognizing the N-terminus of the *TRPV6* protein was used. Na^+^/K^+^ ATPase and β-actin were used for normalizing the amount of protein taken for western blot analysis. **(E)** Cytosolic Ca^2+^ as assessed by a ratiometric Fura-2 assay in cells transiently transfected with the empty vector, or plasmids harboring either the wild type or mutant *TRPV6* cDNA. No significant differences in the basal or resting Ca^2+^ levels were observed in cells expressing either the wild type or p.V492Tfs*136 *TRPV6* when compared to the empty vector. Expression of wild type *TRPV6* in cells significantly increased Ca^2+^ uptake on exposure to 2 mM Ca^2+^, whereas p.V492Tfs*136 failed to increase Ca^2+^ uptake. Each data point represents the average of 16 measurements taken from different regions of a microplate well. The data shown is the mean ± standard deviation of data from a single experiment, which is representative of experiments performed twice. *p* values shown were calculated using one-way ANOVA with Welch’s correction.

We found a novel mutation (2 bp deletion) in the transient receptor potential cation channel subfamily V member 6, *TRPV6* (c.1474_1475delGT; p.V492Tfs*136), in the affected trio (Table 2). The deletion was confirmed by Sanger sequencing (Figure 1C), and found in a heterozygous state in the affected individuals. The frameshift that resulted from the 2 bp deletion that lies in the ion transport domain (Figure 2A) would generate an altered amino acid sequence from p.V492 such that the mutant protein extends to p.626 position before reaching a stop codon. The sequence beyond V492 bears no resemblance to the wild type *TRPV6* (Figure 2B).

**TABLE 2:** List of mutations present in the affected patients (FSD), their allele frequencies in the different populations, including data from recent 1000 Indian genomes (*Indigenome*).

Status	SNP ID	Variation type	Gene	Amino acid modification	MAF score (gnomAD)	MAF score (1000G)	SAS _MAF score (gnomAD)	SAS_MAF score (1000G)	IndiGen_MAF 1000G
Novel Mutation	NA	Frameshift (Del)	*TRPV6*	p.V492Tfs*136	NA	NA	NA	NA	NA
Clinically relevant variants	rs1042636	Missense (SNV)	*CASR*	Arg990Gly	.0899	.20647	.2277	.2638	.2490
	rs7955866	Missense (SNV)	*FGF23*	Thr239Met	.1139	.147364	.1138	.1196	.1205

SNV, single nucleotide variation; MAF, minor allele frequency; SAS, South Asian; NA, not available.

### Functionally defective variant in transient receptor potential vanilloid subfamily member 6 (*TRPV6*) is associated with hereditary pancreatitis

*TRPV6* is a constitutively active Ca^2+^ channel (Venkatachalam and Montell, 2007) and belongs to the vanilloid subfamily of transient receptor potential channels. It is the most selective TRP channel for Ca^2+^ (Nilius and Flockerzi, 2014), and is expressed in certain epithelial cells, where it has been proposed to play a role in Ca^2+^reabsorption. The precise physiological function of *TRPV6* in most tissues remains largely unknown [reviewed in Fecher-Trost et al. (2017)]. In humans, the *TRPV6* gene is expressed in pancreatic ductal cells (Segerstolpe et al., 2016), where it is thought to be involved in Ca^2+^ uptake from the duct lumen (Masamune et al., 2020). *TRPV6* mutations have very recently been identified in pancreatitis patients of Japanese, Chinese, and European ethnicity, but there have been no reports from the South Asian population (Masamune et al., 2020; Zou et al., 2020; Oracz et al., 2021; Hamada et al., 2022). A preliminary characterization of the few variants so far reported to be associated with pancreatitis demonstrated deficient calcium transport or decreased levels of protein expression (Masamune et al., 2020; Zou et al., 2020; Oracz et al., 2021; Hamada et al., 2022). Examination of the structure of human *TRPV6* predicted that as a consequence of the two base pair deletion seen in our patients, the truncation would delete the central Ca^2+^ channel forming domain (Figure 2C).

We examined the expression and localization of wild type (WT) or p.V492Tfs*136 *TRPV6* in HEK293T cells following transient transfection of plasmids containing the corresponding cDNAs. Wild type *TRPV6* (Mr ∼80 kDa) was localized to both the membrane and cytosolic fractions (Figure 2D). However, the mutant protein, which migrated at a predicted molecular weight of ∼60 kDa, was not detected in the membrane fraction, but localized to the cytosol (Figure 2D).

Following transfection of wild type or p.V492Tfs*136 *TRPV6* cDNAs in HEK293T cells, cytosolic Ca^2+^ levels were measured by a ratiometric Fura-2 assay. While no differences in the resting Ca^2+^ levels were evident in cells expressing wild type or p.V492Tfs*136 *TRPV6* as compared to the empty vector control, significantly higher Ca^2+^ uptake was observed in cells expressing wild type *TRPV6* in response to 2 mM Ca^2+^ than that seen in cells transfected with an empty vector. Cells transfected with the p.V492Tfs*136 variant, however, failed to display increased Ca^2+^ uptake in response to 2 mM Ca^2+^ (Figure 2E). Together, these studies suggest that the p.V492Tfs*136 *TRPV6* variant is a loss-of- function protein which is not localized to the cell surface and defective in Ca^2+^ transport.

### Variants in additional genes in the affected trio that could contribute to clinical presentation

Genetic mutations associated with pancreatitis could be influenced by variants in additional disease-modifying genes. The calcium-sensing receptor (CASR) is a plasma membrane protein, expressed to moderately high levels in the endocrine cells in the pancreas (Baron et al., 2016). *CASR*, encoded by *CASR*, regulates intracellular calcium levels based on extracellular Ca^2+^ concentrations. We noted a gain-of-function variant in *CASR* (p.R990G) in patients that was absent in the mother (Supplementary Figure S1 and Table 2) and present in a heterozygous state. The *CASR* R990G variant has been reported to be significantly associated with chronic pancreatitis and in subjects who reported moderate alcohol consumption (Muddana et al., 2008). This variant is found to be common in different populations (Table 2), and increased Ca^2+^ levels in cells in response to lower concentrations of extracellular calcium in comparison with wild type *CASR* (Vezzoli et al., 2007). However, both loss and gain-of-function mutations of the *CASR* gene have been associated with pancreatitis (Hasan et al., 2018), and recent studies even suggest that variants do not modify the risk for chronic pancreatitis (Takats et al., 2021).

Of clinical relevance in our study is a variant we identified in fibroblast growth factor 23 (*FGF23*; Supplementary Figure S1). *FGF23* is a bone-derived endocrine hormone that orchestrates phosphate and calcium homeostasis, along with parathyroid hormone (PTH) and 1,25-dihydroxy vitamin D. *FGF23* binds to its receptor complex FGFR/Klotho in the kidney (Rodríguez, 2020). *FGF23* regulates Vitamin D biosynthesis in two ways. First, it inhibits 25-hydroxyvitamin D-1α-hydroxylase (*CYP27B1*), which converts 25-hydroxyvitamin D into biologically active 1,25-dihydroxyvitamin D and second, stimulates 1,25-dihydroxyvitamin D_3_ 24-hydroxylase (*CYP24A1*), which accelerates 1,25-dihydroxyvitamin D inactivation (and degradation of 25-hydroxyvitamin D). Together, these *FGF23*-mediated effects decrease the concentrations of active 1,25-dihydroxyvitamin D, leading to a reduced intestinal absorption of calcium and, to a lesser extent, intestinal phosphate absorption [reviewed in Edmonston and Wolf (2020)]. The mutation in *FGF23* (rs7955866; p.T239M) present in our patients has been proposed to stabilize the *FGF23* protein and cause enhanced downstream signaling (Rendina et al., 2012). Although *FGF23* is not expressed in the human pancreas (Segerstolpe et al., 2016), elevated serum *FGF23* levels could lead to low serum Vitamin D levels seen in the affected patients in the family. Indeed, deficiency in Vitamin D levels has been shown to predict the severity of pancreatitis (Huh et al., 2019).

### *Trpv6* is upregulated in caerulein-induced acute pancreatitis in mice

**FIGURE 3:**
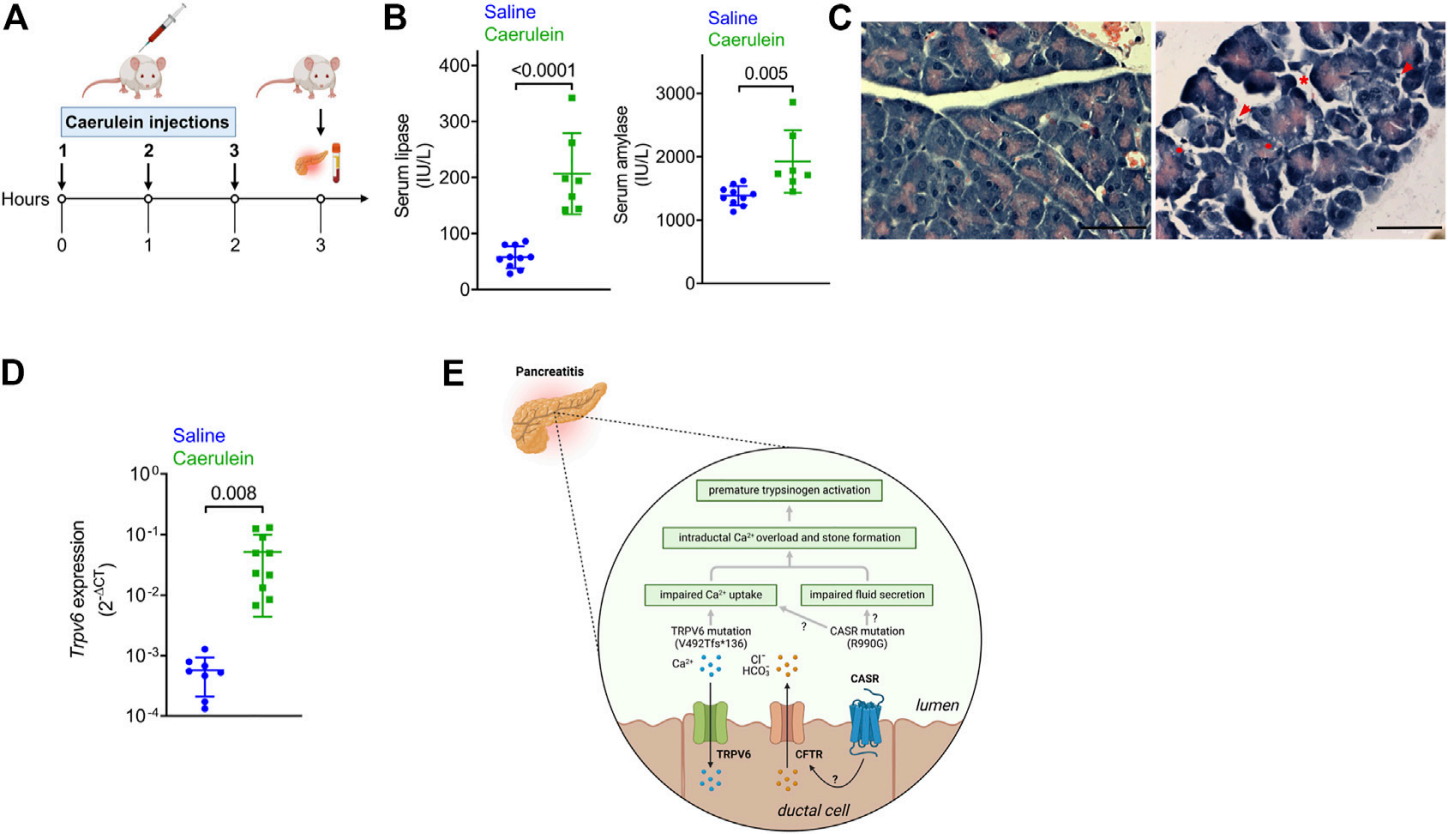
Expression of the genes in the pancreas following caerulein-induced early onset acute pancreatitis. **(A)** Schematic depicting the experimental protocol for caerulein-induced acute pancreatitis. The Figure was created with Biorender. **(B)** Serum lipase (left) and amylase (right) levels in mice injected with saline or caerulin. Data shown are mean ± standard deviation from experiments performed at least thrice and data points represent individual mice. **(C)** Representative images of hematoxylin and eosin-stained pancreatic tissue sections from mice injected with saline or caerulein. Arrows indicated infiltrated immune cells. * denotes edema and circles represent vacuolized acinar cells. Scale bar = 50 μm. **(D)**. Real time quantitative PCR (RTqPCR) analysis of *Trpv6* expression following caerulean-induced pancreatitis. Each point on the scatter plots represents data from a single mouse. The data are presented as the mean SD, and *p* values were calculated using the Student’s t-test. **(E)**. Proposed mechanism underlying the role of defective *TRPV6* in pancreatitis. Loss of function of *TRPV6* in patients harboring the frameshift mutation can lead to impaired Ca^2+^ uptake from the ductal lumen. This can cause increased ductal Ca^2+^ concentrations, premature activation of trypsinogen (the product of *PRSS1*) and subsequent cellular damage and autodigestion. *CASR* is proposed to respond to high Ca^2+^ concentrations in the ductal lumen by triggering fluid and ion secretion, possibly through regulation of CFTR activity. The activating *CASR* p.R990G mutation sensitizes the receptor to Ca^2+^ thereby increasing fluid and ion secretion into the ducts leading to edema. The *CASR* p.R990G mutation may also cause impaired Ca^2+^ uptake in the pancreatic duct, as has been reported in the renal tubules. Based on this model, the cumulative effects of both *TRPV6* and *CASR* mutations may cause Ca^2+^ overload in the ductal lumen and formation of stones, both of which are known risk factors for pancreatitis. The Figure was created with Biorender.

Expression of a mutant protein may have more deleterious effects if its levels are modulated during pancreatic inflammation. We therefore monitored transcript levels of *TRPV6* in the mouse pancreas following onset of acute pancreatitis in a well-established model of cerulein-induced pancreatitis (Figure 3A) (Zhao et al., 2018). Instead of the six to seven hourly injections usually administered to these mice, we sacrificed mice after three injections, to monitor changes that occur during the onset of acute pancreatitis. Caerulein administration significantly increased serum amylase and lipase activities compared with control mice that received saline (Figure 3B). Histological analysis of the sections from control and cerulein-treated mice showed increased pancreatic edema, intracellular vacuoles in acinar cells and increased infiltration of inflammatory cells in caerulein-treated mice (Figure 3C).

*Trpv6* was significantly upregulated in early stages of caerulein-induced acute pancreatitis (Figure 3D). Notably, while *CASR* is reported to be expressed in the human pancreas (Baron et al., 2016), *Casr* and *Fgf23*, were not detected in RTqPCR (data not shown), potentially indicating species differences. Translating the observation that induction of pancreatitis in mice robustly induced *TRPV6* expression, we propose that the aberrant activity of mutant *TRPV6* may initiate pancreatitis and its severity due to increased expression during active disease.

## Discussion

*PRSS1*-linked HP is more common in North American, North-East Asian, and North European ethnicities, but is rare or absent in African or Indian populations (Chandak et al., 2004). We did not observe mutations or CNVs in *PRSS1*, or mutations in other genes that are associated with pancreatitis, in this study. Instead, we report for the first time, a novel heterozygous frameshift mutation caused by a 2bp deletion in *TRPV6* (p.V492Tfs*136) in the affected trio, along with a heterozygous *CASR* variation (p.R990G).

Calcium signaling plays a pivotal role in the regulated secretion of the digestive enzymes, and fluids by the exocrine pancreas (Gerasimenko et al., 2018). Dysregulated Ca^2+^ levels in the pancreatic acinar cells are fundamental to the pathogenesis of pancreatitis by causing premature activation of PRSS1 (Krüger et al., 2000), ER stress (Sah et al., 2014) or hampered zymogen granule trafficking in the acinar cells (Messenger et al., 2014). *TRPV6*, a Ca^2+^ channel is thought to be involved in the reuptake of calcium from the duct lumen in the pancreas (Masamune et al., 2020). Defective *TRPV6* that is seen in our family can result in elevated levels of Ca^2+^ in the duct lumen and consequent Ca^2+^ dysregulation in the cytoplasm. Elevated Ca^2+^ levels in the duct may cause premature activation of PRSS1 in the duct leading to ductal stress and cell damage (Figure 3E). Recent studies that have reported an association of *TRPV6* mutations with chronic pancreatitis in multinational, multiethnic cohorts strengthen a causative link between mutant *TRPV6* and pancreatitis (Masamune et al., 2020; Zou et al., 2020; Oracz et al., 2021; Hamada et al., 2022).

The gain of function *CASR* mutation p.R990G (Vezzoli et al., 2007), present in our patients makes the receptor more sensitive to extracellular Ca^2+^ and could lead to high intracellular Ca^2+^ levels (Vezzoli et al., 2007). *CASR* is expressed in the endocrine pancreas and to lower levels in pancreatic ductal cells in humans (Baron et al., 2016), suggesting that mutations in both *TRPV6* and *CASR* may have additive or synergistic effects in the ducts. Although the precise role of *CASR* in pancreatic ductal cells is unknown, it has been proposed that it responds to high calcium concentrations in the juice by triggering fluid and ion secretion and ductal flushing, possibly through regulation of CFTR activity, thereby preventing stone formation and pancreatitis (Mayerle et al., 2019) (Figure 3E). Furthermore, the p.R990G variant has been shown to inhibit Ca^2+^ uptake from the lumen in the renal tubule leading to hypercalciuria and formation of kidney stones (Vezzoli et al., 2007). A similar pathomechanism may exist in pancreatic ductal cells, such that individuals harboring the 990G variant allele would show decreased Ca^2+^ uptake (Figure 3E). This combined effect of mutations in *TRPV6* and *CASR* would lead to intra-ductal Ca^2+^ overload, an established causal factor for premature activation of pancreatic enzymes and pancreatitis (Figure 3E). Furthermore, there is a strong relationship between increased intraductal Ca^2+^ concentration, precipitation of calcium salts in the duct lumen, and stone formation, which could independently predispose to pancreatitis (Figure 3E) (Mayerle et al., 2019).

Our data also shows that *TRPV6* mRNA is upregulated in mice during caerulein-induced, early-onset acute pancreatitis. Masamune et al. (2020) has reported that caerulein-induced pancreatitis was exacerbated in *TRPV6* ^mut/mut^ mice compared to wild type mice suggesting that functional *TRPV6* is important for protection against pancreatitis.

Low Vitamin D levels in the serum have been shown to predict the severity of chronic pancreatitis (Huh et al., 2019). Our patients also show low levels of serum Vitamin D, perhaps contributed by the activating and stabilizing mutation in *FGF23* (p.T239M) as discussed earlier. An increase in serum *FGF23* level is also associated with elevated blood phosphate *in vivo* [reviewed in Noonan and White (2019)]. *FGF23* knock out mice experiments revealed that the paramount physiological function of *FGF23* is its role in the regulation of Vitamin D levels *via CYP27B1*, and *CYP24A1* transcription, and not its phosphaturic function (Erben, 2018). Therefore, the *FGF23* p.T239M mutation present in our patients might explain low vitamin D levels and marginally higher levels of phosphate in the serum of patient S and D.

In conclusion, we report a novel frameshift mutation in *TRPV6* in an Indian family with hereditary pancreatitis that renders the mutant protein inactive. We also find a relevant mutation in *CASR* in this family. Unlike earlier reports on HP where mutations in *PRSS1* and *SPINK1* have been the focus so far, our results suggest that the disease can be caused by mutations, which may act in synergy. Upregulation of the *TRPV6* wild-type allele in heterozygous carriers by vitamin D administration has been reported earlier (Nilius and Flockerzi, 2014) and could provide a promising therapeutic approach for pancreatitis research in the future (Sahin-Tóth, 2020).

## Materials and methods

### Subjects

Three members of a family, Father (46 years), Son (11 years), and Daughter (08 years) presenting episodes of recurrent acute pancreatitis were recruited at Christian Medical College (CMC), Vellore, Tamil Nadu, India. Unlike the father, who had the first episode at 12 years of age and has progressed to the chronic stage of disease, children had an early onset (less than 5 years). The unaffected mother of the children aged 38 years was taken as a control for the study.

### Sample collection

The study was reviewed and approved by the Institutional Ethics Committees of both Christian Medical College (CMC) Vellore (IRB No. 11254), and the Indian Institute of Science (IISc), Bangalore (IHEC No: 2-23012019). 2–3 mL of whole blood was collected from all four subjects by trained nurses at CMC Vellore in EDTA-coated vials and stored in −80°C before it was shipped in dry ice to the IISc, Bangalore for further analysis. The medical and family history of all the subjects was also recorded. Written informed consent was taken from all the subjects.

### Whole exome analysis

DNA was isolated from the whole blood using QIAamp^®^ DNA Blood Mini Kit (Qiagen). Genomic DNA from each subject was subjected to whole-exome analysis (Clevergene, Bangalore). One microgram of genomic DNA was taken for library preparation using Next DNA II Library preparation Kit for *Illumina* ^®^ (New England Biolabs). The genomic library was enriched by using xGEN Research panel V1.0 (Integrated DNA Technologies) as per the manufacturer’s protocol before sequencing on the Illumina HiSeq platform to generate 2 × 150 bp reads. The quality check of sequence data was done using FastQC and MultiQC (Babraham-Bioinformatics-FastQC; Ewels et al., 2016) software. The adapter sequences, low-quality reads were removed using *fast p* (Chen et al., 2018). To call variants, Genome Analysis Tool Kit (GTAK) best practices were followed. In brief, the quality trimmed reads were mapped to the human reference genome build hg19 with the use of BWA and MEM algorithm (Li and Durbin, 2009). PCR duplicates were removed from the mapped reads using Picard tools (Broad Institute). The base qualities were recalibrated using GATK v4.0.12.0 (Van der Auwera et al., 2013) and Genome Variant Call Formats (GVCFs) were generated for each sample. The GVCFs were combined before variants were called. The variant quality scores were recalibrated to obtain the probability that an SNP is a true genetic variant and not a sequencing or data processing artifact.

All annotations for the pass filtered variants were extracted from Variant Effect Predictor (VEP) (McLaren et al., 2016) (ensemble.org). All the missense variants present in the affected trio (FSD), not present in the mother, with gAD allele frequency of ≤.01 were considered for further analysis. The variants that had a Combined Annotation Dependent Depletion (CADD) score of ≥20 were retained (Rentzsch et al., 2019). In the genetic analysis, only the variants that presented high genotype confidence and were predicted to be deleterious by five algorithms, namely SIFT (Kumar et al., 2009), PolyPhen-2 (Adzhubei et al., 2010), MetaSVM (Kim et al., 2017), MCAP (Jagadeesh et al., 2016), and MutationTaster (Schwarz et al., 2010) were considered for further analysis. Novel mutations in the affected trio, where allele frequency and prediction of being deleterious were not available on VEP, were retained during the analysis. The allele frequencies of the variants that came from the final analysis were also checked in the Indian 1000 genome (Indigenome project) (Jain et al., 2021). All genetic variants of interest were further validated by Sanger sequencing.

### Copy number variant analysis

One microgram of gDNA was subjected to an array-based CNV analysis (InfiniumOmni5Exome-4v1-3, Illumina) performed by Macrogen Inc., South Korea.

### Bioinformatics

Wild type and mutant protein sequence of *TRPV6* was obtained *in silico* by translating the wild type and mutant (having desired 2 bp deletion) mRNA (NM_018646.6) sequence using the translation tool of *expasy.org.* Sequence alignment of both the protein sequences was done using *embnet.vital-it.ch.* The published cryo-EM structure of human *TRPV6* (McGoldrick et al., 2018) (PDB ID: 6BO8) was used to identify regions deleted in the mutant *TRPV6*. RNA sequencing data sets for the study included (a) E-MTAB-5061 with single cell RNA-seq analysis of human pancreas from healthy individuals and (b) GSE84133 with single cell RNA-seq analysis of mouse and human pancreas.

### Cell culture, plasmids and antibodies

HEK293T cells were obtained from ATCC and grown in DMEM/high glucose/sodium pyruvate containing 10% fetal bovine serum in a 5% CO2 incubator at 37°C. The *TRPV6* cDNA in pLenti6.3/V5-Dest backbone was obtained from the DNASU plasmid repository (HsCD00860923). *TRPV6* cDNA was then subcloned into pBKS vector using *SpeI* and *XhoI* sites for mutagenesis. Mutant *TRPV6* was generated by site-directed mutagenesis using two primers 5′ CAG​CGG​GGA​GGT​GAC​CCA​TGT​CCT​TTG​C 3′ and 5 GCA​AAG​GAC​ATG​GGT​CAC​CTC​CCC​GCT​G 3′. The *TRPV6* cDNA with 2bp desired deletion was cloned back into the pLenti6.3/V5-Dest backbone. The loss of the *KpnI* restriction site resulting from the mutation was used for screening, and the plasmid was confirmed by sequencing. Membrane and cytosolic proteins from HEK293T cells transiently transfected with empty vector, WT and mutant *TRPV6* cDNA constructs were extracted and Western blotting was performed as previously described (Mishra et al., 2021). Antibodies used were anti-*TRPV6* from Abclonal (A16128) that detects amino acids 1–100 of human *TRPV6*, anti-Na^+^/K^+^ ATPase from Abcam (ab76020), anti-β-actin from Cell Signalling Technology (4970S), and HRP conjugated anti-rabbit IgG from Sigma-Aldrich (A0545).

### Measurement of cytosolic Ca^2+^ changes

Cytosolic Ca^2+^ was assessed as previously described (Prasad et al., 2019), with modifications. Briefly, cells were washed with PBS and loaded with Fura-2 AM (Invitrogen F1221) at 1 μg/mL in calcium-containing buffer (2 mM CaCl_2_, 126 mM NaCl, 4.5 mM KCl, 2 mM MgCl_2_, 10 mM glucose, 20 mM HEPES, pH 7.4). After 30 min at room temperature, cells were washed three times for 5 minutes in calcium-containing buffer without Fura-2 AM and incubated for another 30 min to allow for Fura-2 AM de-esterification. Cells were replaced with calcium-free buffer (126 mM NaCl, 4.5 mM KCl, 2 mM MgCl_2_, 10 mM glucose, 20 mM HEPES, pH 7.4), and resting calcium levels and calcium uptake after the addition of 2 mM Ca^2+^ were measured by exciting cells at 340 nm and 380 nm and emission captured at 510 nm using a microplate reader (Tecan Infinite M200 PRO). Ratios of 340/380 nm emissions were was calculated and plotted.

### Caerulein induced early-onset, acute pancreatitis

All procedures were carried out in agreement with the Control and Supervision Rules, 1998 of Ministry of Environment and Forest Act (Government of India), and the Institutional Animal Ethics Committee of the Indian Institute of Science (Approval CAF/Ethics/547/2017). All animals were bred and housed in the same vivarium. Mice were housed in a clean air facility in multiple cages and separated on the basis of sex and genotype. The temperature was maintained at 22°C ± 2°C, humidity was maintained at 55% ± 10%, and the mice were maintained on a 12-h light/dark cycle. Mice had access to laboratory chow and water *ad libitum*. Chow was procured from Altromin International (Germany) and contained ∼24% protein, 6% oil, and 3% dietary fibers.

Pancreatitis was induced in overnight fasted 8–10 weeks old female C57BL6/N mice by the administration of three repeated intraperitoneal hourly injections of caerulein (*Merck*; 50 µg/kg body weight) (Reed and Gorelick, 2014). Control mice were administered phosphate buffered saline. Mice were sacrificed 1 hour after the last injection. The pancreas was quickly isolated and stored in pre-cooled RNA-later (Merck) for 6–7 h at 4°C before storing at −80°C. RNA was prepared from ∼20 mg of the tissue using the RNAeasy RNA extraction Kit (Qiagen) following manufacturer’s instructions. The expression profile of mutant genes of the affected trio was assessed by quantitative real-time PCR using SYBR Premix Ex Taq (Tli RNase H Plus; Takara Bio) on a CFX96 Touch real-time PCR detection system (Bio-Rad). The housekeeping gene glyceraldehyde 3-phosphate dehydrogenase (*Gapdh*) was used for normalization. Primers used for real-time PCR are *Trpv6* forward primer: 5′- TCT​GCA​GAT​GGT​TCC​ACA​GAC-3′; *Trpv6* reverse primer: 5′-AGG​GCT​GCT​ATG​TGA​AGT​GC-3′, *Gapdh* forward primer: 5′-CAA​CTC​CCT​CAA​GAT​TGT​CAG​CAA-3′; and *Gapdh* reverse primer: 5′-GGC​ATG​GAC​TGT​GGT​CAT​GA-3′).

## Data Availability

The datasets for this article are not publicly available due to concerns regarding participant/patient anonymity. Requests to access the datasets should be directed to the corresponding author.
