# Supplementary material for: A novel frameshift mutation in TRPV6 is associated with hereditary pancreatitis
Source: Front Genet. 2023 Jan 9;13:1058057. doi: 10.3389/fgene.2022.1058057 (PMC9868559; doi:10.3389/fgene.2022.1058057)
Supplement: Supplementary file 1 [file Image1.pdf]

Supplemental Information Figure 1

A novel frameshift mutation in TRPV6 is associated with hereditary pancreatitis

Shah et al

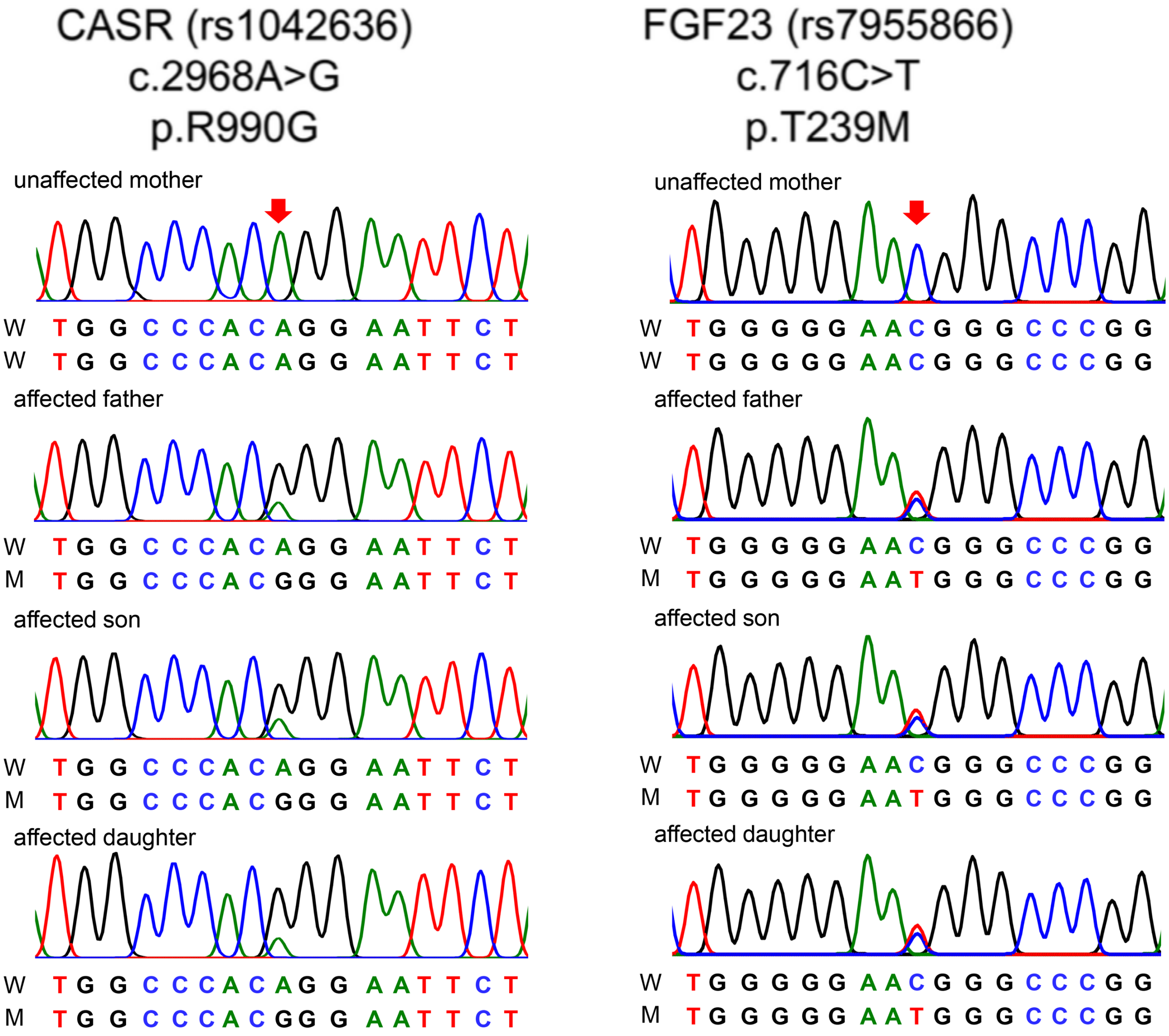

Figure S1: Clinically relevant mutations in CASR and FGF23
Chromatogram representing c.2968A>G in *CASR* (left) and c.716C>T in *FGF23* (right) in the affected trio as a heterozygous state.
